# Do visual and step height factors cause imbalance during bipedal and unipedal stances? A plantar pressure perspective

**DOI:** 10.3389/fbioe.2023.1253056

**Published:** 2023-08-17

**Authors:** Panjing Guo, Duoduo Wang, Yumin Li, Ruiqin Wang, Haoran Xu, Jia Han, Jie Lyu

**Affiliations:** ^1^ College of Rehabilitation Sciences, Shanghai University of Medicine and Health Sciences, Shanghai, China; ^2^ School of Health Science and Engineering, University of Shanghai for Science and Technology, Shanghai, China

**Keywords:** plantar pressure, static balance, kinematic parameters, dynamic parameters, center of plantar pressure, posture control

## Abstract

**Objective:** The plantar pressure analysis technique was used to explore the static balance ability and stability of healthy adult males under the influence of visual and step height factors during bipedal and unipedal stances.

**Methods:** Thirty healthy adult males volunteered for the study. Experiments used the F-scan plantar pressure analysis insoles to carry out with eyes open (EO) and eyes closed (EC) at four different step heights. The plantar pressure data were recorded for 10 s and pre-processed to derive kinematic and dynamic parameters.

**Results:** For unipedal stance, most of kinematic parameters of the subjects’ right and left feet were significantly greater when the eyes were closed compared to the EO condition and increased with step height. The differences in toe load between right and left feet, open and closed eyes were extremely statistically significant (*p* < 0.001). The differences in midfoot load between the EO and EC conditions were statistically significant (*p* = 0.024) and extremely statistically significant between the right and left feet (*p* < 0.001). The difference in rearfoot load between EO and EC conditions was extremely statistically significant (*p* < 0.001) and statistically significant (*p* = 0.002) between the right and left feet. For bipedal stance, most of kinematic parameters of the subjects’ EO and EC conditions were statistically significant between the right and left feet and increased with step height. The overall load’s difference between EO and EC states was statistically significant (*p* = 0.003) for both feet. The overall load’s difference between the right and left feet was extremely statistically significant (*p* < 0.001) in the EC state. The differences between the right and left feet of the forefoot and rearfoot load with EO and EC suggested that the right foot had a smaller forefoot load, but a larger rearfoot load than the left foot (*p* < 0.001). The differences between the forefoot and rearfoot load of the subjects’ both feet with EO and EC were extremely statistically significant (*p* < 0.001).

**Conclusion:** Both visual input and step height factors, even the dominant foot, act on kinematic and dynamic parameters that affect the maintenance of static balance ability.

## 1 Introduction

Balance is an ability to maintain physical stability throughout human daily activities (Lin et al., 2016). Vision, proprioception, and vestibular sensation play a crucial role in postural control in the static stance (Fitzpatrick and McCloskey, 1994), which provide peripheral sensory information. Then it transmitted to the central nervous system for information processing, integration, and finally through visual positioning and motor control to regulate the body’s center of gravity position (Germano et al., 2016). Loss of just one of the three sensory systems can cause deterioration in postural stability (Magnusson et al., 1990). Among these, visual input is an important factor affecting balance control, and some studies have shown that visual input can be used to improve control of posture for balance training (Pellegrino et al., 2017; Fischetti et al., 2020) and even boost other rehabilitation outcomes (Xia et al., 2022). Moreover, the adjustment in the standing posture depends on the motor control of the foot, but there is still no clear conclusion on the mechanism through which the foot controls the balance function and the link between visual input and foot regulation (Giagazoglou et al., 2009). Therefore, the present study was designed to elucidate the effect of visual input on static balance function in terms of two parameters: kinematic and dynamic, using plantar pressure analysis.

Poor balance often leads to fall injuries. Falls are the second most common cause of fatal unintentional injuries in the world (Jiao et al., 2023). For most people, the fear of falling is a phenomenon that occurs when standing in extreme situations such as on the edge of a cliff, a tall step or a high building. People who experience this dread forgo many essential daily tasks, which negatively affects their physical ability, independence, level of activity, and quality of life in terms of their health (Tinetti et al., 1994; Cumming et al., 2000; Brouwer et al., 2004). Over 25% of ankle sprains requiring hospital treatment, according to a survey of 100 emergency department visits at hospitals over a 4-year period, were caused by falls down stairs (Waterman et al., 2010). Furthermore, in a study conducted on older individuals’ falls and the conditions surrounding them, 49 percent of outdoor falls that resulted in injuries occurred on a sidewalk, street, or curb (Duckham et al., 2013). The prevalence of ankle sprains brought on by falls from stairs and curbs, as well as the fact that up to 75% of people still feel discomfort and/or sprains after their initial injuries, indicating that falls during transition step negotiation are a serious public health issue (Hubbard and Wikstrom, 2010). Although it has been suggested that lower curbs may lessen the likelihood that individuals will be hurt when crossing the street (Dommès et al., 2015), to our knowledge, no studies have specifically examined differences in static human balance for steps of different lower heights. The majority of step research done to date has mostly focused on the kinematics and moments of the ankle, knee, and hip joints during serial stair climb and descent (Andriacchi et al., 1980; McFadyen and Winter 1988; Zachazewski et al., 1993; Mian et al., 2007; Protopapadaki et al., 2007). In addition, we also discovered that prior research has concentrated on falls between people who have already sustained a primary injury from a fall as opposed to those who have not. Therefore, there are few research on the risk and foot pressure before falls, despite the fact that preventing falls is the most crucial step in preventing them (Yan et al., 2023). However, plantar pressure is a crucial component of standing and walking. Detecting and analyzing it can increase our awareness of potential hazards before a fall (Niu et al., 2019). Accordingly, the present study also aimed to elucidate the effect of different step heights on static balance function, including two parameters: kinematic and dynamic by plantar pressure analysis.

Ultimately, we raise a question whether plantar pressure analysis can be used for balance assessment. At this stage, the assessment of balance function is mainly based on qualitative assessment by clinical assessment scales and quantitative assessment by balance function measurement systems, such as the Berg Balance Scale, the Stand and Walk Test, the Pro-Kin Balance System, the Tertrax Balance Test System, etc (Lin et al., 2020). However, in recent years, with the popularization of the plantar pressure system, some studies have used it for the quantitative analysis of balance function (Ema et al., 2016; Howcroft et al., 2016; Parsons et al., 2016; Dibai-Filho et al., 2017; Hamed et al., 2018; Patti et al., 2018). The present experiment was similarly designed to verify whether plantar pressure analysis could be applied to balance function assessment, using this technique to assess postural control in healthy young males under the influence of different visual and step height factors.

## 2 Materials and methods

### 2.1 Participants

The inclusion criteria were:① Age between 18 and 30 years old; ② In good physical and mental health with normal motor function; ③ Deny any prior history of related diseases causing balance abnormalities, such as cerebrovascular disease, multiple sclerosis, bilateral lower limb fractures, discomfort in the lower limbs, long and short legs, arthritis, *etc.*; ④ Deny any ongoing use of medicines that alter balancing function on a regular basis; ⑤ Sign the informed consent form voluntarily.

Thirty healthy male students (23.9 ± 1.19 years old; 76.01 ± 11.86 kg body mass; 176.9 ± 6.14 cm height; 7-8 UK foot size; all dominant feet were right feet (Gribble et al., 2007; Lin et al., 2009)) from University of Shanghai for Science and Technology were recruited as volunteers. All volunteers completed the Berg Balance Scale test before the experiment, which is widely used and has high credibility and validity (Berg et al., 1992). All volunteers scored 52.63 ± 1.49 greater than 41, indicating that they all had no balance dysfunction with a good consistency (Downs et al., 2014). All subjects participated voluntarily and signed the basic information questionnaire and informed consent form.

### 2.2 General experimental procedures

#### 2.2.1 Pre-test preparation

##### 2.2.1.1 Environmental preparation

**FIGURE 1:**
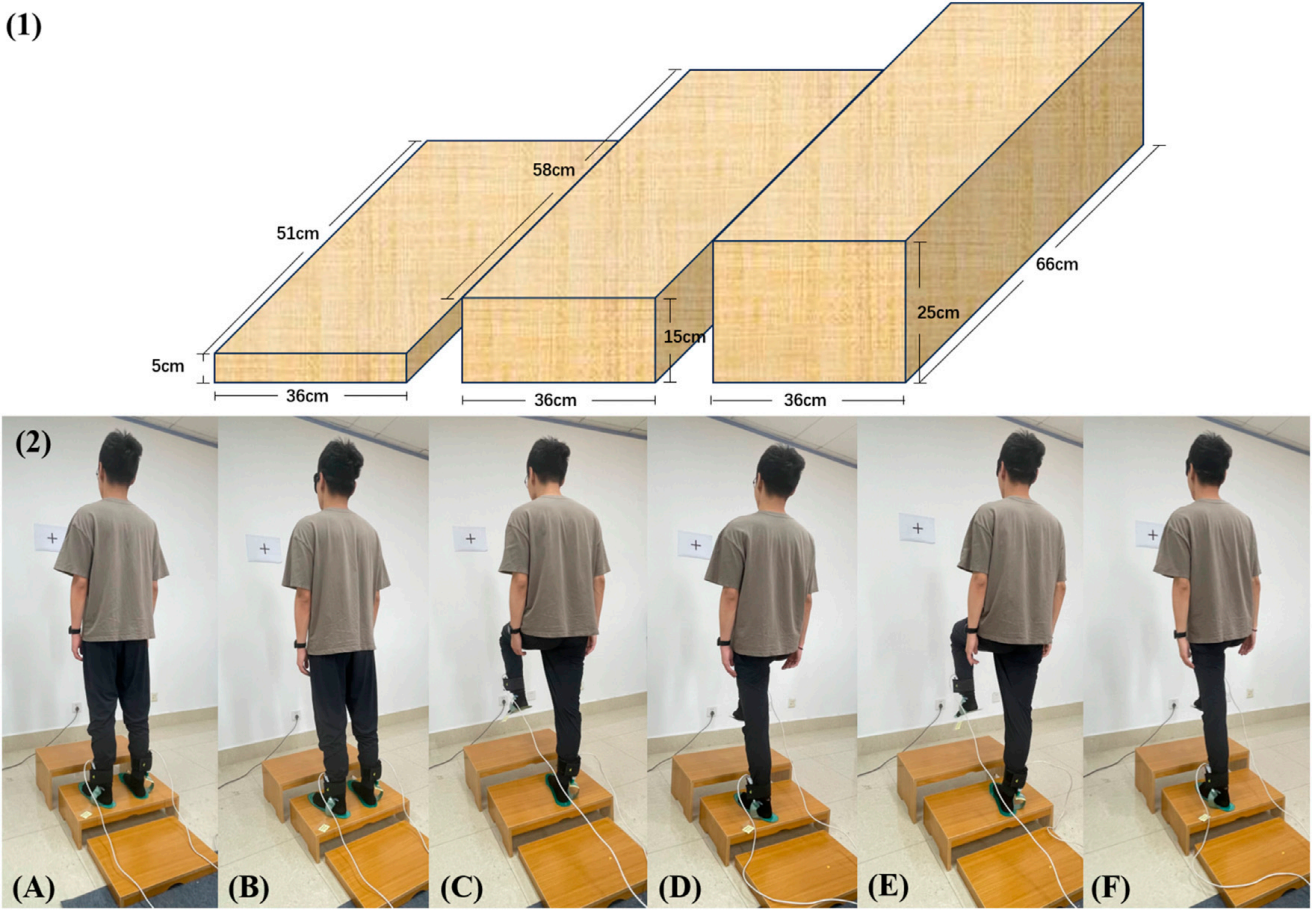
(1) The design of the steps of different heights. (2) The whole experiment with 15 cm step height as an example. **(A)** bipedal standing with eyes open; **(B)** bipedal standing with eyes closed; **(C)** unipedal (right foot is grounding) standing with eyes open; **(D)** unipedal (left foot is grounding) standing with eyes open; **(E)** unipedal (right foot is grounding) standing with eyes closed; **(F)** unipedal (left foot is grounding) standing with eyes closed.

To avoid interference from outside sources, the experiment should be conducted in a quiet space. To lessen the impact of head and body shaking on the experimental results, the plantar pressure analysis test device was positioned approximately 1.5 m from a blank wall with a height-adjustable black "+" marker of about 10 cm × 10 cm, whose position was adjusted in accordance with the subject’s height so that it was level with the subject’s eyes. The subject swung his pelvis in four directions: anterior-posterior (AP) and medial-lateral (ML) so that the subject’s plantar pressure was evenly distributed. The experiment was conducted on a flat surface with three steps as shown in Figure 1 1) (The heights could be adjusted by 5, 15, and 25 cm. The length, width, and height of the three steps are 51 × 36 × 5 cm; 58 × 36 × 15 cm; 66 × 36 × 25 cm), which were hard and level without sacrificing generality. The 0 cm step height is a standard standing on the ground and the 5, 15, and 25 cm step heights encompass standardized curb and building code stair heights and a step that is 2.5 cm above current US Federal Highway Administration guidelines (Gerstle et al., 2017). The steps’ heights were changed to simulate the height factor of a healthy adult. The eyes of the subject will alternately open and close to mimic the visual effect. Subjects would be informed of the height of the steps during the experiment. Last but not least, the subject’s actual standing foot served as the data-gathering object.

To finish the experiment with eyes open (EO) and eyes closed (EC), the subjects were placed at four different heights: 0 cm, 5 cm, 15 cm, and 25 cm, respectively. Two different experimental groups were performed as shown in Figure 1 2). Experimental group 1: ① bipedal standing with eyes open, ② bipedal standing with eyes closed; Experimental group 2: ① unipedal (right foot is grounding) standing with eyes open, ② unipedal (left foot is grounding) standing with eyes open, ③ unipedal (right foot is grounding) standing with eyes closed, ④ unipedal (left foot is grounding) standing with eyes closed.

##### 2.2.1.2 Equipment preparation

The F-scan plantar pressure analysis system (Tekscan, Boston, MA, United States), which permits real-time monitoring and feedback of the pressure at the “foot-ground interface” throughout the whole gait support phase, is the force-measuring insole. The same insole, whose size could be altered, was used for testing all participants. Its thickness was 0.15 mm, covering 4 piezoresistive sensors per 1 cm^2^, with the sensor measuring a range of 0 kPa–862 kPa. The sampling frequency was set at 50 Hz. The data of each sensor on the insole were output and exported from the plantar pressure analysis system at each instant to process and analyze the subjects’ plantar pressure data in different experimental situations during the test period.

The force-measuring insole will develop creases under stress due to the softness of the shoe lining material, reducing the accuracy of the data when placed in the shoe. Accordingly, the subjects removed their shoes during the test, and a uniform cotton sock was used as the medium to securely adhere the force-measuring insole to the subject’s toe, arch, and heel with regular double-sided adhesive. This was done to prevent the relative positions of the test insole and the subject’s foot from shifting during the preparation activities and the standing process. The uniformity of the measured data positions was thus guaranteed.

##### 2.2.1.3 Participants preparation

Subjects refrain from indulging in strenuous exercise the day before and the day of the test to minimize the impact on the experiment. The volunteers were instructed to take a 5-min break before the test to acclimate to the environment and de-stress so that psychological factors would not affect the test results.

#### 2.2.2 Start of the test

1. Eyes-open state monitoring: Each subject was instructed to look 1.5 m ahead of the black "+" marker while standing in an upright position on a flat ground or a height platform. Plantar pressure data were collected in the subject’s eye-open state. After around 5 s of stability, the individual started to record plantar pressure data for 10 s.
2. Eye-closed state monitoring: Each subject was instructed in an upright position with their eyes closed on a flat surface or a step with height. After around 5 seconds of stability, the individual started to record plantar pressure data for 10 s.
3. Bipedal stance: Subjects were instructed to stand in the upright position with both of their feet on the ground.
4. Unipedal stance: Subjects were instructed to stand unipedally with the right foot and unipedally with the left foot, with the opposite foot raised upward throughout the unipedal stance, not touching the ground and not generating plantar pressure data.
5. To lessen the impact of acoustic stimulation or stress on the results, the subjects were not informed that the data were being recorded. Throughout the experiment, a second tester stood behind the participant to prevent a fall.
6. The procedure of the plantar pressure test is provided below. 1) Two calibrations of F-scan system equipment; 2) Create and enter a new subject’s basic information on the F-scan software; 3) Attach the test insole to the thumb, arch, and heel of the subject’s foot with ordinary double-sided tape, and try to stand for 5 min; 4) After a 2-min rest, let the subject complete the movement according to the experimental method to collect data and repeat the experiment three times, with the same process as above. 5) Rename all the experimental data of the subject and export two files, one for the kinematic data and the other for the dynamic data.

#### 2.2.3 Plantar pressure data process

The plantar pressure data is exported from the F-scan plantar pressure analysis system, containing the unprocessed plantar pressure center data and plantar pressure distribution data for each frame. The center of plantar pressure (COP) oscillation is the most reliable parameter for assessing postural stability, and changes in the position of the center of pressure can reflect static balance during standing (Pinsault and Vuillerme, 2009; Paillard and Noé, 2015). In this paper, a custom Python code (Pycharm Community Edition 2022.2, JetBrains s. r.o., Prague, Czech Republic) was used for data processing and exporting the applicable parameter metrics.

The primary categories of the indicators are kinematic parameters and dynamic parameters. Kinematic parameters included COP-ML adjustment velocity (mm/s), COP-AP adjustment velocity (mm/s), COP adjustment velocity (mm/s), 95% confidence circle area (mm^2^), ML range (mm), AP range (mm), maximum swing (mm), minimum swing (mm), mean X, mean Y; dynamic parameters include overall load, toe load, forefoot load, midfoot load and rearfoot load of the right and left bilateral lower limbs. The *X*-axis (mean X) and *Y*-axis (mean Y) data were used to pinpoint the center of plantar pressure (COP). These are also obtained by a custom Python program with the relevant formulas (Singh et al., 2009; Bickley et al., 2019; Quijoux et al., 2021), which are shown in Table 1.

**TABLE 1:** Formulas related to kinematic parameters.

Kinematic parameters	Formula
COP-AP adjustment path (mm)	$COP-AP\;adjustment\;path=1/N\sum_{n=1}^{N}\left|AP\left[n\right]\right|$
COP-ML adjustment path (mm)	$COP-ML\;adjustment\;path=1/N\sum_{n=1}^{N}\left|ML\left[n\right]\right|$
COP adjustment path (mm)	$COP\;adjustment\;path=1/N{\sum_{n=1}^{N}\left[AP{\left[n\right]}^{2}+ML{\left[n\right]}^{2}\right]}^{1/2}$
COP Total adjustment time (s)	COP Total adjustment time=T
COP-AP adjustment velocity (mm/s)	$COP-AP\;adjustment\;velocity=1/T\sum_{n=1}^{N-1}\left|AP\left[n+1\right]-AP\left[n\right]\right|$
COP-ML adjustment velocity (mm/s)	$COP-ML\;adjustment\;velocity=1/T\sum_{n=1}^{N-1}\left|ML\left[n+1\right]-ML\left[n\right]\right|$
COP adjustment velocity (mm/s)	$COP\;adjustment\;velocity=1/T{\sum_{n=1}^{N-1}\left[{\left(AP\left[n+1\right]-AP\left[n\right]\right)}^{2}+{\left(ML\left[n+1\right]-ML\left[n\right]\right)}^{2}\right]}^{1/2}$
95% confidence circle area (mm^2^)	$Mean\;Distance=1/N{\sum_{n=1}^{N}\left[AP{\left[n\right]}^{2}+ML{\left[n\right]}^{2}\right]}^{1/2}$
	$RMS\;Distance={\left[1/N\sum_{n=1}^{N}\left[AP{\left[n\right]}^{2}+ML{\left[n\right]}^{2}\right]\right]}^{1/2}$
	$95\%\;confidence\;circle\;area=\pi {\left(MDIST+1.645{\left[RDIST^{2}-MDIST^{2}\right]}^{1/2}\right)}^{2}$
ML range (mm)	$ML\;range=\max_{1\leq n\leq m\leq N}\left|ML\left[n\right]-ML\left[m\right]\right|$
AP range (mm)	$AP\;range=\max_{1\leq n\leq m\leq N}\left|AP\left[n\right]-AP\left[m\right]\right|$
Maximum swing (mm)	${Maximum\;swing=\max}_{1\leq n\leq N-1}{\left[{\left(AP\left[n+1\right]-AP\left[n\right]\right)}^{2}+{\left(ML\left[n+1\right]-ML\left[n\right]\right)}^{2}\right]}^{1/2}$
Minimum swing (mm)	$Minimum\;swing=\min_{1\leq n\leq N-1}{\left[{\left(AP\left[n+1\right]-AP\left[n\right]\right)}^{2}+{\left(ML\left[n+1\right]-ML\left[n\right]\right)}^{2}\right]}^{1/2}$
Mean X (mm)	$Mean\;X=1/N\sum_{n=1}^{N}ML_{n}$
Mean Y (mm)	$Mean\;Y=1/N\sum_{n=1}^{N}AP_{n}$

### 2.3 Statistical analysis

Data were analyzed using SPSS Statistics (version 26.0; IBM, Chicago, IL, United States) and Excel 2016 (Microsoft, Chagrin Falls, OHIO, United States), and scatter plots were plotted using GraphPad Prism 9 (Microsoft, Chagrin Falls, OHIO, United States). All data are expressed as mean ± standard deviation (M ± SD). Unipedal and bipedal plantar pressure center data, Unipedal plantar pressure distribution data: Firstly, a three-way analysis of variance (ANOVA) for repeated measures was used. A 2 × 2 × 4 (vision × dominant foot × step height) within subject ANOVA was performed to investigate the influence of vision, dominant foot, step height and their interaction for all kinematic parameters and dynamic parameters. In cases where the ANOVA indicated a significant effect (*p* < 0.05), two-way ANOVA for repeated measures was used to examine the main effects and the interaction of two factors on the above parameters. Bonferroni correction was used for *post hoc* multiple comparisons. Bipedal plantar pressure distribution data: paired *t*-test was used for normal distribution and the Wilcoxon rank sum test was used for skewed distribution. We set the α level *a priori* at 0.05. *p* < 0.05 was used to indicate statistical difference and *p* < 0.001 was used to indicate extremely significant statistical difference.

## 3 Results

### 3.1 Center of plantar pressure (COP) data (kinematic parameters)

#### 3.1.1 COP data on unipedal stance

A three-way ANOVA for repeated measures based on vision, dominant foot and step height for the aforementioned kinematic parameters showed that there was no statistical difference in the factor of the dominant foot on unipedal stance for the aforementioned kinematic parameters. Therefore, a two-way ANOVA for repeated measures was performed again for the effects of vision and step height, as shown in Table 2, and there were no significant differences in any of the interactions.

**TABLE 2:** Comparison of COP data on unipedal stance for different visual factors at different step heights.

	0 cm step		5 cm step		15 cm step		25 cm step	
	EO	EC	EO	EC	EO	EC	EO	EC
**Right foot**								
COP-ML adjustment velocity (mm/s)	18.50 ± 4.81	45.91 ± 16.16	19.62 ± 6.05	48.39 ± 14.78	22.18 ± 4.51	49.41 ± 15.09	21.45 ± 5.77	49.95 ± 14.15***
COP-AP adjustment velocity (mm/s)	21.45 ± 8.39	55.48 ± 23.61	23.66 ± 8.35	75.40 ± 71.09	27.19 ± 9.05	66.98 ± 36.85	30.31 ± 20.16	75.26 ± 59.83***
COP adjustment velocity (mm/s)	31.44 ± 9.06	80.62 ± 28.46	33.97 ± 9.31	100.78 ± 67.98	38.74 ± 8.90	92.75 ± 38.36	41.05 ± 19.82	101.30 ± 56.75***
95% confidence circle area (mm^2^)	833.11 ± 681.07	2618.88 ± 1659.87	1123.13 ± 1015.96	3485.55 ± 3961.47	1372.77 ± 1173.38	3173.98 ± 2560.72	1336.88 ± 1291.91	3290.28 ± 3164.00***
ML range (mm)	21.47 ± 5.32	39.27 ± 9.25	22.01 ± 6.34	40.05 ± 6.23	25.99 ± 7.12	40.38 ± 8.79	23.41 ± 6.30	40.49 ± 9.16***
AP range (mm)	34.68 ± 14.77	70.97 ± 33.88	40.38 ± 20.07	76.58 ± 37.52	43.29 ± 17.62	76.98 ± 33.62	49.1 ± 28.54	74.95 ± 34.64***
Maximum swing (mm)	3.35 ± 1.57	11.41 ± 10.11	3.90 ± 1.89	17.89 ± 27.83	4.10 ± 1.19	15.00 ± 15.97	5.83 ± 6.13	14.03 ± 12.73***
Minimum swing (mm)	0.02 ± 0.01	0.04 ± 0.02	0.02 ± 0.01	0.04 ± 0.03	0.02 ± 0.01	0.04 ± 0.03	0.02 ± 0.02	0.04 ± 0.03***
Mean X (mm)	58.15 ± 3.49	58.82 ± 5.54	57.64 ± 5.24	56.23 ± 5.51	57.19 ± 5.09	56.07 ± 5.50	56.31 ± 6.09	56.46 ± 5.53
Mean Y (mm)	141.38 ± 13.75	147.73 ± 15.88	137.12 ± 19.11	145.92 ± 14.44	139.52 ± 18.48	144.14 ± 19.83	138.09 ± 18.65	145.04 ± 18.19*
**Left foot**								
COP-ML adjustment velocity (mm/s)	18.21 ± 4.92	44.08 ± 11.69	19.44 ± 4.73	45.35 ± 13.05	22.85 ± 6.44	50.20 ± 9.57	23.82 ± 7.28	50.55 ± 13.43***^###^
COP-AP adjustment velocity (mm/s)	23.28 ± 11.83	65.27 ± 34.71	23.03 ± 11.32	66.26 ± 36.29	29.99 ± 27.94	74.58 ± 45.44	38.60 ± 53.15	86.61 ± 88.64***
COP adjustment velocity (mm/s)	32.65 ± 12.64	87.44 ± 39.98	33.20 ± 12.36	88.99 ± 37.36	41.98 ± 28.65	100.14 ± 43.62	50.93 ± 52.87	111.87 ± 85.55***^#^
95% confidence circle area (mm^2^)	895.99 ± 930.23	3295.92 ± 2989.81	845.97 ± 736.13	3006.71 ± 2210.12	1155.69 ± 1857.87	3857.99 ± 3653.01	1748.49 ± 3375.01	3682.59 ± 4622.27***
ML range (mm)	21.20 ± 5.86	38.97 ± 9.28	22.34 ± 5.52	39.74 ± 8.96	23.48 ± 6.31	41.53 ± 5.53	23.53 ± 6.93	39.48 ± 7.32***
AP range (mm)	35.30 ± 20.11	72.77 ± 36.78	36.50 ± 18.16	70.72 ± 35.98	39.64 ± 29.73	79.50 ± 37.72	45.42 ± 39.91	72.81 ± 43.66***
Maximum swing (mm)	3.90 ± 2.66	14.35 ± 14.79	3.74 ± 1.77	12.09 ± 11.64	5.99 ± 8.83	13.88 ± 12.97	9.21 ± 19.46	16.30 ± 18.20***
Minimum swing (mm)	0.01 ± 0.01	0.04 ± 0.02	0.01 ± 0.01	0.04 ± 0.02	0.02 ± 0.01	0.04 ± 0.02	0.02 ± 0.01	0.04 ± 0.03***
Mean X (mm)	45.30 ± 4.34	46.42 ± 5.91	47.24 ± 4.00	48.22 ± 4.85	45.86 ± 4.65	49.34 ± 6.50	46.45 ± 5.63	48.24 ± 5.79*
Mean Y (mm)	138.60 ± 16.52	145.45 ± 14.05	142.18 ± 16.50	147.73 ± 15.69	141.27 ± 15.44	147.56 ± 10.87	138.39 ± 14.29	148.13 ± 13.66***

*p < 0.05 indicate statistical difference between different visual factors with EO, and EC; ***p < 0.001 indicate extremely significant statistical difference between different visual factors with EO, and EC.

^#^ *p* < 0.05 indicate statistical difference between different step heights of 0 cm, 5 cm, 15 cm, and 25 cm; ^###^ *p* < 0.001 indicate extremely significant statistical difference between different step heights of 0 cm, 5 cm, 15 cm, and 25 cm.

As shown in Table 2, comparing the EO and EC states, the differences in COP-ML adjustment velocity, COP-AP adjustment velocity, COP adjustment velocity, 95% confidence circle area (mm^2^), ML range (mm), AP range (mm), maximum swing (mm), minimum swing (mm) between the right and left feet and Mean Y of the left foot were extremely statistically significant (*p* < 0.001). The differences in Mean Y (*p* = 0.003) of the right foot and Mean X (*p* = 0.007) of the left foot were statistically significant. These kinematic parameters were significantly increased when the eyes were closed compared to the open-eye condition. When compared to the EO situation, all of these kinematic parameters were much higher on the EC situation. The differences in the COP adjustment velocity (*p* = 0.029) of the left foot were statistically significant, and the differences in the COP-ML adjustment velocity (*p* < 0.001) of the left foot were extremely statistically significant when comparing the four step heights of 0cm, 5cm, 15cm, and 25 cm. All of these kinematic parameters increased with increasing height.

#### 3.1.2 Comparison of scatter diagrams of COP data on unipedal stance

**FIGURE 2:**
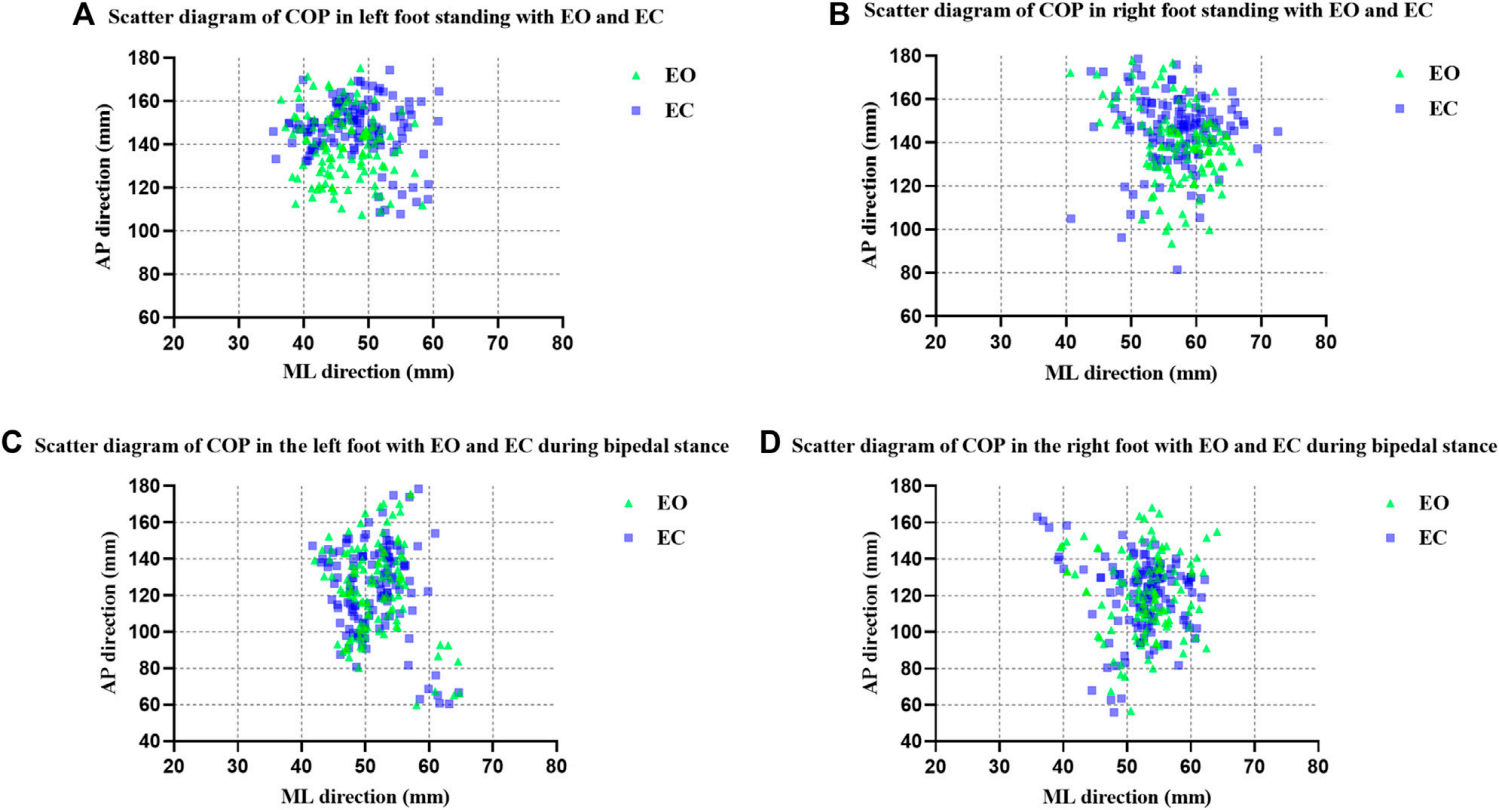
Scatter diagram of COP **(A)** in left foot standing with EO and EC; **(B)** in right foot standing with EO and EC; **(C)** in the left foot with EO and EC during bipedal stance; **(D)** in the right foot with EO and EC during bipedal stance.

Scatter plots of COP data on unipedal stance for left and right feet by mean X and mean Y were made. The mean *X*-axis and *Y*-axis coordinates of the center of plantar pressure during the test were calculated and the results were obtained as the mean X and mean Y. The COP was further localized based on the scatter plot distribution of the COP in Figures 2A,B which showed that the COP was distributed more towards the mid-anterior part of the foot in the EC condition than in the EO condition.

#### 3.1.3 COP data on bipedal stance

A three-way ANOVA for repeated measures based on vision, dominant foot and step height for the aforementioned kinematic parameters showed that there was no statistical difference in the factor of vision on bipedal stance for the aforementioned kinematic parameters. Therefore, a two-way ANOVA for repeated measures was performed again for the effects of dominant foot and step height, as shown in Table 3, and there were no significant differences in any of the interactions.

**TABLE 3:** Comparison of COP data on bipedal stance between the right foot and left foot at different step heights.

	0 cm step		5 cm step		15 cm step		25 cm step	
	Right foot	Left foot	Right foot	Left foot	Right foot	Left foot	Right foot	Left foot
**EO**								
COP-ML adjustment velocity (mm/s)	2.46 ± 1.60	2.44 ± 1.95	3.27 ± 1.70	2.49 ± 1.68	3.53 ± 1.62	2.64 ± 1.87	4.48 ± 2.59	2.71 ± 1.42^#^^$$$^
COP-AP adjustment velocity (mm/s)	11.70 ± 8.81	11.78 ± 7.33	15.5 ± 8.83	12.32 ± 8.25	15.52 ± 5.64	12.35 ± 8.11	17.59 ± 7.91	14.53 ± 7.51^#$^
COP adjustment velocity (mm/s)	12.33 ± 9.12	12.39 ± 7.77	16.28 ± 9.16	12.95 ± 8.62	16.42 ± 5.94	13.02 ± 8.49	18.82 ± 8.58	15.17 ± 7.77^#$^
95% confidence circle area (mm^2^)	588.39 ± 1236.85	484.19 ± 618.75	595.90 ± 1338.71	839.06 ± 2180.87	468.86 ± 448.64	569.45 ± 789.45	855.24 ± 1312.69	1128.89 ± 1710.36
ML range (mm)	2.53 ± 1.57	2.21 ± 2.30	2.82 ± 1.63	2.38 ± 2.65	2.61 ± 1.51	1.99 ± 1.18	3.51 ± 1.91	2.78 ± 1.90^$^
AP range (mm)	25.44 ± 19.19	24.84 ± 16.85	26.30 ± 20.56	28.20 ± 24.52	26.06 ± 11.30	27.04 ± 16.61	33.25 ± 21.31	36.58 ± 23.71^#^
Maximum swing (mm)	2.04 ± 2.34	1.81 ± 1.42	2.96 ± 3.92	1.95 ± 1.83	2.58 ± 1.86	1.91 ± 1.28	2.70 ± 1.54	2.64 ± 1.92
Minimum swing (mm)	0.002 ± 0.004	0.004 ± 0.007	0.003 ± 0.004	0.002 ± 0.004	0.004 ± 0.005	0.002 ± 0.004	0.005 ± 0.006	0.003 ± 0.004
Mean X (mm)	51.23 ± 3.90	49.16 ± 3.53	53.62 ± 5.16	51.55 ± 3.68	55.25 ± 4.67	52.52 ± 4.50	54.97 ± 5.54	53.12 ± 5.21^###$$$^
Mean Y (mm)	131.07 ± 18.93	129.53 ± 27.27	112.89 ± 22.15	120.36 ± 23.18	115.71 ± 19.76	123.89 ± 27.55	120.89 ± 24.16	120.34 ± 24.02^#^
**EC**								
COP-ML adjustment velocity (mm/s)	2.57 ± 1.26	2.25 ± 1.47	3.36 ± 1.44	2.24 ± 1.21	3.38 ± 1.25	2.35 ± 1.71	3.95 ± 1.59	2.50 ± 1.31^#$$$^
COP-AP adjustment velocity (mm/s)	12.85 ± 6.25	12.83 ± 7.52	16.45 ± 6.67	12.62 ± 6.79	17.42 ± 7.64	14.45 ± 8.11	18.39 ± 7.65	14.41 ± 6.94^#$^
COP adjustment velocity (mm/s)	13.49 ± 6.48	13.35 ± 7.79	17.22 ± 6.92	13.15 ± 7.00	18.20 ± 7.86	14.94 ± 8.45	19.36 ± 7.91	15.00 ± 7.17^#$^
95% confidence circle area (mm^2^)	935.93 ± 1491.39	1074.51 ± 2002.06	839.21 ± 1105.53	852.03 ± 1255.97	1164.21 ± 1837.69	1316.18 ± 2732.26	1713.86 ± 4227.93	1456.77 ± 3816.6
ML range (mm)	2.62 ± 1.49	2.11 ± 1.80	2.77 ± 1.54	2.10 ± 1.25	2.83 ± 1.76	2.80 ± 4.18	3.52 ± 2.21	2.77 ± 2.38
AP range (mm)	31.28 ± 18.88	31.50 ± 22.57	33.43 ± 20.69	31.69 ± 18.96	37.51 ± 26.96	37.43 ± 30.43	37.59 ± 30.05	34.99 ± 26.96
Maximum swing (mm)	2.06 ± 1.55	1.94 ± 1.43	2.50 ± 1.33	1.86 ± 1.38	3.05 ± 1.76	2.52 ± 1.85	3.00 ± 2.55	2.23 ± 1.81^$^
Minimum swing (mm)	0.003 ± 0.005	0.002 ± 0.004	0.003 ± 0.005	0.001 ± 0.003	0.003 ± 0.005	0.001 ± 0.003	0.004 ± 0.005	0.003 ± 0.005^$^
Mean X (mm)	52.51 ± 4.29	49.70 ± 4.51	54.05 ± 6.19	51.81 ± 4.59	55.05 ± 4.59	51.80 ± 4.17	55.67 ± 5.30	52.85 ± 4.80^#$$$^
Mean Y (mm)	121.62 ± 19.72	126.42 ± 25.38	118.80 ± 22.22	125.23 ± 26.46	118.79 ± 21.66	120.14 ± 24.71	119.20 ± 19.80	120.92 ± 21.54

^#^ *p* < 0.05 indicate statistical difference between different step heights of 0 cm, 5 cm, 15 cm, and 25 cm; ^###^ *p* < 0.001 indicate extremely significant statistical difference between different step heights of 0 cm, 5 cm, 15 cm, and 25 cm.

^$^ *p* < 0.05 indicate statistical difference between right foot and left foot; ^$$$^ *p* < 0.001 indicate extremely significant statistical difference between right foot and left foot.

As shown in Table 3, comparing the two states of the right and left foot, the differences in COP-ML adjustment velocity (*p* < 0.001) and mean X (*p* < 0.001) on the EO situation were extremely statistically significant; the differences in COP-AP adjustment velocity (*p* = 0.022), COP adjustment velocity (*p* = 0.016), and ML range (mm) (*p* = 0.030) were statistically significant. The differences in COP-ML adjustment velocity (*p* < 0.001), and mean X (*p* < 0.001) on the EC situation were extremely statistically significant; the differences in COP-AP adjustment velocity (*p* = 0.004), COP adjustment velocity (*p* = 0.002), maximum swing (mm) (*p* = 0.025), and minimum swing (mm) (*p* = 0.005) were statistically significant. All of these kinematic parameters were significantly greater in the right foot compared to the left foot. The differences in COP-ML adjustment velocity (*p* = 0.008), COP-AP adjustment velocity (*p* = 0.030), COP adjustment velocity (*p* = 0.025), AP range (mm) (*p* = 0.032), and mean Y (*p* = 0.011) on the EO situation were statistically significant; the difference in mean X (*p* < 0.001) was extremely statistically significant when comparing the four heights of 0 cm, 5 cm, 15 cm, and 25 cm. The differences of COP-ML adjustment velocity (*p* = 0.019), COP-AP adjustment velocity (*p* = 0.033), COP adjustment velocity (*p* = 0.031), and mean X (*p* = 0.004) on the EC situation were statistically significant. All of these kinematic parameters increased with increasing height.

#### 3.1.4 Comparison of scatter diagrams of COP data on bipedal stance

Scatter plots of COP data on bipedal stance for left and right feet by mean X and mean Y were made. The COP was further localized based on the scatter plot distribution of the COP in Figures 2C,D, which showed that there was no significant difference in the distribution of the COP in the EC condition compared to the EO condition.

### 3.2 Plantar pressure distribution data (dynamic parameters)

#### 3.2.1 Plantar pressure distribution data on unipedal stance

Firstly, we compared the effects of vision, dominant foot and step height on the plantar pressure distribution data by a three-way ANOVA for repeated measures. According to the results, the main effect of different heights was not statistically significant for these dynamic parameters, and the remaining effects of dominant foot and vision were statistically significant. The differences were analyzed by repeated two-way ANOVA. As a result, the final interaction effects were not of significant difference.

As shown in Table 4, the differences in toe load on the both right and left feet and visual factors were extremely statistically significant (*p* < 0.001). There was also a greater load on the right foot than on the left foot and an increase in load in the EC condition than in the EO condition. There was a statistical difference (*p* = 0.024) between midfoot load in the EO and EC conditions, and an extremely significant statistical difference (*p* < 0.001) between the right and left feet. There was an increase in load in the EC condition than in the EO condition, and a greater load on the right foot than on the left foot. The difference in rearfoot load (*p* < 0.001) was extremely statistically significant for the EO and EC conditions, and statistically significant (*p* = 0.002) for the right and left feet. The rearfoot load was significantly smaller in the EC condition than in the EO condition, and it was larger on the right foot than on the left foot.

**TABLE 4:** Comparison of plantar pressure distribution data with different visual factors on the left and right foot stance.

Items	Right foot		Left foot	
	EO (M ± SD,%)	EC (M ± SD,%)	EO (M ± SD,%)	EC (M ± SD,%)
Toe load	5.80 ± 4.05	8.73 ± 5.49	3.52 ± 3.43	6.10 ± 4.61***^$$$^
Midfoot load	48.29 ± 10.69	50.32 ± 9.92	53.57 ± 11.83	56.07 ± 11.15*^$$$^
Rearfoot load	45.92 ± 10.74	40.95 ± 10.65	42.91 ± 11.11	37.83 ± 9.62***^$^

*p < 0.05 indicate statistical difference between different visual factors with EO and EC; ***p < 0.001 indicate extremely significant statistical difference between different visual factors with EO and EC.

^$^ *p* < 0.05 indicate statistical difference between right foot and left foot; ^$$$^ *p* < 0.001 indicate extremely significant statistical difference between right foot and left foot.

#### 3.2.2 Plantar pressure distribution data on bipedal stance

##### 3.2.2.1 Comparison of plantar pressure distribution parameters between the EO and EC states of the right and left feet

Firstly, we compared the effects of vision, dominant foot and step height on the plantar pressure distribution data by a three-way ANOVA for repeated measures. According to the results, the main effect of different heights was not statistically significant for these dynamic parameters, and the remaining main effects of dominant foot and vision were statistically significant. Then, the correlation between them was analyzed by paired *t*-test.

As shown in Table 5, the difference between the overall load of the right and left foot in the EO and EC state was statistically significant (*p* = 0.003), with the overall load of the right foot increasing and the left foot decreasing in the EO state. The differences between the forefoot load and rearfoot load of the right and left feet in the EO and EC conditions were not statistically significant.

**TABLE 5:** Comparison of plantar pressure distribution data with different visual factors when standing on different feet.

Items	Location	EO (M ± SD,%)	EC (M ± SD,%)	*t* value	*p*-Value
Overall load	Right Foot	49.29 ± 7.11	53.10 ± 6.71	3.09	0.003
	Left Foot	50.71 ± 7.11	46.90 ± 6.71	−3.09	0.003
Forefoot load	Right Foot	36.49 ± 16.51	36.66 ± 13.82	−0.08	0.935
	Left Foot	42.86 ± 15.94	42.82 ± 16.55	0.02	0.983
Rearfoot load	Right Foot	63.51 ± 16.51	63.34 ± 13.82	0.08	0.935
	Left Foot	57.14 ± 15.94	57.19 ± 16.55	−0.02	0.983

##### 3.2.2.2 Comparison of plantar pressure distribution parameters in both feet with different visual factors

As shown in Table 6, the difference in the overall load between the left and right foot was not statistically significant when the eyes were open (*p* = 0.445). However, with eyes closed, the difference in the overall load of the left and right foot was extremely statistically significant (*p* < 0.001). And the overall load of the right foot started to increase and the left foot decreased. The difference between the forefoot load and rearfoot load of the right and left feet was extremely statistically significant (*p* < 0.001) when the eyes were open and closed. The forefoot load of the right foot was extremely smaller than that of the left foot, but the rearfoot load of the right foot was similarly larger than that of the left foot.

**TABLE 6:** Comparison of plantar pressure distribution data of the right and left feet with different visual factors.

Items	Status	Right foot (M ± SD,%)	Left foot (M ± SD,%)	*t* value	*p*-Value
Overall load	EO	49.29 ± 7.11	50.71 ± 7.11	−0.77	0.445
	EC	53.10 ± 6.71	46.90 ± 6.71	−3.57	<0.001
Forefoot load	EO	36.49 ± 16.51	42.86 ± 15.94	−4.26	<0.001
	EC	36.66 ± 13.82	42.82 ± 16.55	−4.63	<0.001
Rearfoot load	EO	63.51 ± 16.51	57.14 ± 15.94	4.26	<0.001
	EC	63.34 ± 13.82	57.19 ± 16.55	4.63	<0.001

##### 3.2.2.3 Comparison of plantar pressure distribution parameters between the forefoot and rearfoot in the EO and EC states

As shown in Table 7, the differences in forefoot load and rearfoot load between the right and left foot of the subjects in the EO and EC conditions were extremely statistically significant (*p* < 0.001). The rearfoot load was significantly greater than the forefoot load.

**TABLE 7:** Comparison of plantar pressure distribution parameters between the forefoot and rearfoot in the EO and EC states.

Location	Status	Forefoot load (M ± SD,%)	Rearfoot load (M ± SD,%)	*t* value	*p*-Value
Right Foot	EO	36.49 ± 16.51	63.61 ± 16.51	−6.34	<0.001
	EC	36.66 ± 13.82	63.34 ± 13.82	−7.48	<0.001
Left Foot	EO	42.86 ± 15.94	57.14 ± 15.94	−3.47	<0.001
	EC	42.82 ± 16.55	57.19 ± 16.55	−3.36	0.001

## 4 Discussion

Balance control depends mainly on three elements: visual, vestibular and proprioceptive, which must be integrated and combined with motor and cognitive systems to maintain body stability (Kilby et al., 2017; Takeda et al., 2017; da Silva Pontes et al., 2019). In a normal person standing with EO, proprioceptive input from the lower limbs and visual input play a key role in balance regulation; when the eyes are closed, the vestibular system replaces the visual input by knowing the position and movement of the head to make appropriate adjustments in each body part to maintain body balance (Dos Anjos et al., 2016; Kim et al., 2018).

In addition, different step heights simulate different actual situations, and the adjustment of height may make subjects increase their psychological burden and generate fear of falling, leading to a decrease in static balance. Studies have shown that the main factor affecting balance self-confidence is the balance performance ability (Hatch et al., 2003), and the lack of balance ability will lead to a decrease in self-confidence, which will induce patients to develop the fear of falling (Patil et al., 2013). Fear of falling leads to over-cautiousness, which can affect normal gait characteristics, muscle force and motor function (Li et al., 2002; Hauer et al., 2009; Ayoubi et al., 2015), seriously affecting patients’ daily activities and reducing their quality of life (Murphy et al., 2002; Franchignoni et al., 2005).

In this experiment, visual deprivation was simulated by opening and closing the eyes in two states, and standing at different heights by means of standing on different height steps, but the foot contact surface and body position were not changed to exclude unstable effects on the proprioceptive and vestibular systems. The effects of visual factors and standing height on balance function were discussed from two aspects, kinematics and dynamics.

### 4.1 Variation in unipedal kinematic parameters

Contrary to the EO state, the EC state changes in the aforementioned parameters during unipedal stance suggested that the subject’s right and left feet swung significantly in both the AP and ML directions. These changes in the parameters were correlated with the activity of the center of gravity sensed through the plantar pressure analysis, responding to the degree of balance function maintenance and adjustment. Muir’s study showed that COP-related parameters were significantly worse among those who fell (Muir et al., 2013). It is hypothesized that the amplitude of COP sway is extremely significantly higher in healthy adult males standing on one foot with EC than in the EO state, which is primarily related to balance control after visual deprivation. So people use their vision to anticipate changes that have an impact on performance on balancing tasks and to react to changes that have occurred (Sember et al., 2020). Moreover, there are no differences in unilateral postural stability between the functionally dominant and nondominant lower limbs in a healthy population of young males, which is consistent with most previous studies (Hoffman et al., 1998; Alonso et al., 2011; King and Wang, 2017).

Comparing the four heights of 0cm, 5cm, 15 cm and 25cm, COP-AP adjustment velocity, COP adjustment velocity in the left foot increased with increasing height. It is revealed that height-induced threat increased subjects’ fear and anxiety and decreased confidence (Cleworth et al., 2019). However, the increase in height would make the balance more difficult to control due to the inherent instability of unipedal stance (Adkin and Carpenter, 2018). Combined with the above discussion of unipedal stance, Freeman’s study concluded that strengthening the unipedal exercise is beneficial for limb stability, and therefore, unipedal stance balance has been used extensively to assess balance disorders associated with musculoskeletal injuries (Freeman et al., 1965). Additionally, the unipedal exercise by adjusting the step height is also expected to improve the fear of falling.

### 4.2 Variation in bipedal kinematic parameters

During bipedal stance, compared with the left foot, there is an obviously significant adjustment in the ML direction in the standing state of the right foot with EO and EC. The study by Pizzigalli concluded that certain swaying characteristics of stance posture, particularly in the ML direction, are significantly different from those of falling and non-falling people (Pizzigalli et al., 2016). COP trajectory on the *X*-axis (ML) is a better predictor of falls than the *Y*-axis (AP) (Portnoy et al., 2017). Besides, there is another adjustment in the AP direction in the standing state of the right foot during bipedal stance. The difference between the right and left foot may be due to the dominant side, since the subject’s dominant side in this experiment was the right side, so the difference was reflected in the right foot. Actually, the dominant foot is closely related to the bipedal stance balance, indicating that the dominant foot has a greater contribution to the bipedal stance balance of the lower limbs (Chen et al., 2020). Accordingly, this study suggest that healthy young males use their dominant foot in general to adjust the body’s center of gravity and maintain the balance and stability of body posture by changing the AP position of the dominant foot’s COP, also consistent with the results of plantar pressure distribution data.

The changes in the adjustment of COP not only in the ML direction but also in the AP direction increased significantly with the rise in height when comparing the four heights of 0cm, 5cm, 15cm, and 25 cm in the EO and EC conditions. Visual deprivation does not change the slowing or increasing of COP oscillations during bipedal stance. Cleworth et al. showed that as step height increases, individuals become more psychologically burdened and dread falling from the higher step, which further contributes to the stabilization of the human body through adjustment of the dominant side as the primary and non-dominant side as the secondary (Cleworth et al., 2016).

### 4.3 Variation in unipedal dynamic parameters

Due to visual deprivation, some subjects may increase their grip on their toes when touching the ground with one foot in the EC condition. As a result, the toe load will be much higher in the EC condition (Matsuno et al., 2022). The difference in midfoot and rearfoot loads between the EO and EC conditions could be explained by the fact that people struggle to maintain balance when their eyes are closed and must increase the midfoot load to do so. Additionally, the subject’s increased toe and midfoot loads have contributed to the decrease in rearfoot load in the EC state. Pol et al. reported that the fallers had greater medial midfoot, medial forefoot, and bunion loading (Pol et al., 2021). Zhao further explained that the toes are composed of distal phalanges, middle phalanges and proximal phalanges, and the other four toes have three joints except the big toe. The phalanges are the parts of the plantar with relatively flexible movement characteristics. In standing, walking and other sports behaviors, muscles drive the phalanges to bend to grasp the ground, which can enhance standing and movement stability (Zhao, 2022). It is worth noting that in the upright stance position, toe muscle strength is closely related to walking and balancing posture (Kamasaki et al., 2023). In addition, toe pressure strength in the standing position discriminated the risk of falling in older adults (Kamasaki et al., 2020). In fact, toe pressure strength in the standing position may be one of the important muscular forces among those associated with standing up (Kamasaki et al., 2023).

The difference associated with the right and left feet is speculated to be related to the dominant side and healthy male college students are more accustomed to using the right foot to support and exert force (Zhao, 2022). The pressure load on the right foot’s toes and rearfoot was higher than the load on the left foot. This is due to the fact that the right foot will distribute more force on both sides of the heel and toe and will be comparably more stable, suggesting that there is a tendency for the dominant foot to be more stable than the non-dominant foot in both lower extremities (Chen et al., 2020). Furthermore, the previous study also found significant differences in postural control during unipedal stance between the dominant and non-dominant feet (Promsri et al., 2018).

### 4.4 Variation in bipedal dynamic parameters

The overall load of the right and left feet changed after EC, with the right foot increasing and the left foot decreasing, showing that visual factors influence the ratio of the center of gravity of the right and left foot. So visual factor has an impact on the lateralization of the right and left foot and there is a significant swing in the ML direction (Anker et al., 2008). Forefoot load and rearfoot load did not differ significantly after EC and EO. It means that for healthy young males, there is a subtle change in the pressure after EO and EC, a negligible change in the center of gravity, and a negligible swing in the AP direction during bipedal standing. Previous studies reported the greater contribution of vision to body sway in the AP direction compared with that in the ML direction (Dickstein and Abulaffio, 2000; Carpenter et al., 2001; Huffman et al., 2010) or the sway by visual occlusion occurred both in the AP and ML directions (Sawaguchi et al., 2022). However, the finding we proposed was not in line with these hypotheses; the increase in the swing induced by visual occlusion occurred in the ML direction, not AP. This may be explained by slight differences in the experimental methodology, population of the participants or fatigue factor causing slightly different statistical power.

In the EC condition, the right foot carried a heavier overall load than the left foot. Meanwhile, the center of the body’s gravity progressively shifted and the ratio of the left and right feet started to vary gradually, becoming unstable. The subjects changed the load on the dominant side to attain a balanced state since it is conducted that an increase in the overall load on the right foot is achieved by an adjustment to the dominant side. Moreover, the right foot had less forefoot load than the left foot, whereas the right foot had a higher rearfoot load than the left foot in the EO and EC states. Additionally, the rearfoot load was significantly higher than the forefoot load. Both findings are in line with the findings of earlier studies and are consistent with healthy male adults who prefer their dominant foot and primarily use their rearfoot bearing weight instead of their forefoot when standing for long periods (de Paula Lima et al., 2017).

## 5 Limitations and future directions

This experiment investigates the static balance ability of healthy adult males, but there are also differences in balance function between males and females (Kozinc et al., 2021; Jo et al., 2022), and our research group will conduct experiments with relevant female subjects for the future. Secondly, the differences in static balance between different experimental environments, such as the material of the floor, can be discussed in the future. Moreover, fatigue is also a factor that needs to be considered in this experiment, as studies have shown that the static balance of the human body varies under different states of fatigue (Gebel et al., 2022). Fatigue induced a significant increase in postural oscillations in the ML direction, with no significant effects in the AP direction (da Silva et al., 2022). Therefore, fatigue has the potential to explain the lateralization results in this paper during bipedal stance. So, the role and function of fatigue in the balance control of the human body need to be further explored. Finally, our team would like to work on quantifying the assessment of static balance and would like to use the existing kinematic and dynamic parameters of plantar pressure to quantify the current major balance function scales.

## 6 Conclusion

Visual, step height factors and even dominant foot can lead to static imbalance in bipedal and unipedal stances for healthy young males. In unipedal stance, they adjusted the COP in the AP and ML directions and changed the weight-bearing ratio distribution between the front and rear of the foot to achieve the regulation of balance function under different visual input states. In unipedal/bipedal stance, the adjustment of COP in the directions of AP and ML gradually increases with the increase of standing height, achieving the regulation of balance function under different step heights. They adjusted the position of the COP in the AP and ML directions of the dominant foot and used the heel of the dominant foot as the support point through lateralized load adjustment, realizing the regulation of balance function under bipedal stance. So the kinematic and dynamic parameters analyzed by plantar pressure can be used for the quantitative assessment of the static balance function. Providing a simple and easy-to-use method to objectively evaluate balance function. It is hoped that this will reduce falls at different height steps and provide a way to improve balance and reduce the fear of falling. Furthermore, hoping to provide some help for early diagnosis, quantitative and clinical evaluation, and rehabilitation training of balance ability for patients with pathologic balance dysfunction such as ankle instability (Shao et al., 2022), lower back pain (Wang et al., 2023), and stroke patients (Liu et al., 2023).

## Data Availability

The raw data supporting the conclusions of this article will be made available by the authors, without undue reservation.

## Abbreviations

COP, Center of plantar pressure; AP, Anterior–posterior; ML, Medial–lateral; EO, Eyes open; EC, Eyes closed; M, Mean value; SD, Standard deviation; ANOVA, Analysis of variance.
